# Emerging NO_2_ gas sensing on substitutionally doped Fe on NiWO_4_ SCES insulators

**DOI:** 10.3389/fchem.2024.1480356

**Published:** 2024-11-05

**Authors:** Jong Hyun Lee, Se Hwang Kang, Gi Hyun Park, Min Young Kim, Sanghyun Ji, Ha Eun Choa, Gi Hyeon Han, Jeong Yun Hwang, Seung Yong Lee, Kyu Hyoung Lee

**Affiliations:** ^1^ Department of Materials Science and Engineering, Yonsei University, Seoul, Republic of Korea; ^2^ Material Science and Chemical Engineering Center Industrial Convergence Engineering Group, Institute for Advanced Engineering, Yongin-si, Gyeonggi-do, Republic of Korea; ^3^ Yonsei-KIST Convergence Research Institute, Seoul, Republic of Korea

**Keywords:** metal oxide, NiWO_4_, gas sensor, NO_2_ gas, SCES

## Abstract

In this study, we demonstrate the emergence of NO_2_ gas sensing capabilities in the typically non-active NiWO_4_, a strongly correlated electron system (SCES), by introducing substitutional Fe at the Ni site. NiWO_4_ typically exhibits strong Coulombic repulsion between Ni atoms, resulting in a large band gap of over 3.0 eV and insulating behavior. This correlated behavior is clearly reflected in the significant increase of band gap when considering the Hubbard *U* correction for the cations, bringing the theoretical value closer to the observed value. The single-phase Fe_0.5_Ni_0.5_WO_4_ displays a notable shift in the [NiO_6_] symmetric vibration mode and an increase in magnetization. Additionally, theoretical calculations confirm the preservation of the wide band gap, with the Fe and O levels generated within the band gap. These findings indicate that Fe located in the Ni sites modulate Coulombic repulsion in NiWO_4_ SCES insulators. Unlike the poor gas-sensing performance of intrinsic NiWO_4_, Fe_0.5_Ni_0.5_WO_4_ exhibits a significant NO_2_ response (R_g_/R_a_) of 11 at 200°C than other gases and a limit of detection (LOD) of 46.4 ppb. This study provides a pathway for realizing gas-sensing performance in strongly correlated electron insulators with large band gaps through the introduction of dopant levels at the cation sites.

## 1 Introduction

Strongly correlated electron systems (SCES) are characterized by Coulombic repulsion between neighboring cations within a crystal structure, leading to the development of exotic physical properties (Dagotto, 2005; Jacqueline et al., 2022; Zewei et al., 2018; Sami et al., 2022; Morosan et al., 2012; Bhargava et al., 2020; Watthaisong et al., 2019; Cesare et al., 2021; You et al., 2016; Park et al., 2020). One notable feature of SCES is the presence of a pseudo-band gap, often referred to as the Mott gap, which arises due to strong Coulombic repulsion between cations, as commonly observed in NiO. The size of this pseudo-band gap depends on the Hubbard parameter (*U*) for each cation, as indicated by theoretical calculations. Additionally, SCES typically exhibit magnetic ordering, often in the form of antiparallel spin alignment, with net magnetization being influenced by the Coulombic repulsion between magnetic cations. However, the higher activation energy for electrical transport in SCES insulators, which typically follows the polaronic hopping conduction model, results in a strong insulating behavior, making these materials challenging for a wide range of applications. Consequently, inherited properties governed by Coulombic repulsion in SCES insulators have inspired various strategies aimed at modulating the interactions between cations.

Among the various strategies for tuning SCES properties, we focus on NiWO_4_, which exhibits Coulombic repulsion between Ni cations similar to the Mott-insulator behavior observed in NiO (Han et al., 2024; Lee et al., 2024). Theoretical calculations show that a pseudo-gap develops as the U value increases, reaching nearly 6 eV of U_Ni_, a value that aligns with the experimentally measured band gap obtained from UV-vis spectroscopy. Additionally, calculation results are supported by the neutron diffraction results of NiWO_4_, confirming the antiparallel spin ordering between Ni cations (Prosnikov et al., 2017). However, NiWO_4_ possesses a monoclinic crystal structure, which has lower symmetry compared to the cubic structure of NiO, and it includes tungsten as an additional component. This provides an extra degree of freedom for tailoring the correlation effects, making it a promising candidate for various applications. For instance, substituting vanadium (V) into the Ni sites, which reduces the Coulombic repulsion strength between cations, could enhance chemical adsorption sites, suggesting the potential of NiWO_4_ for diverse catalytic applications (Suh et al., 2024).

The chemoresistive type of gas sensor exemplifies an intersectional application of physical and chemical properties (Wang et al., 2018; Kim et al., 2023; Dey, 2018; Choi et al., 2021; Yamazoe, 2005; Lee et al., 2023; Jin et al., 2022; Chen et al., 2013; Franco et al., 2022; Ji et al., 2019). For optimal performance, such a sensor must ensure high and monotonic adsorption of gas molecules at specific sites on the material surface. This adsorption leads to the development of surface depletion or accumulation layers due to the exchange of charge carriers between the analyte gases and the material surface. The type of gas molecules (whether reducing or oxidizing) and the conduction type of the material (n-type or p-type) influence the change in resistance after gas exposure. The resistance then returns to its initial value once the analyte gas molecules desorb from the material surface. To enhance gas sensing functionality, we substitutionally introduced Fe mixed with Ni into NiWO_4_, which initially exhibited poor gas-sensing performance. The Fe cation level, positioned within the band gap and modulating the weaker Coulombic repulsion, resulted in improved NO_2_ gas sensing and selectivity. These findings present a strategy for expanding the potential applications of SCES materials beyond gas sensing, which can be achieved by tailoring Coulombic repulsion.

## 2 Materials and methods

### 2.1 Material synthesis

The NiWO_4_ and Fe_0.5_Ni_0.5_WO_4_ powders were synthesized using a solid-state reaction. High-purity Fe_2_O_3_ (Kojundo Chemical Lab, 99.9%), NiO (Kojundo Chemical Lab, 99.97%), and WO_3_ (Kojundo Chemical Lab, 99.9%) powders were mixed following the nominal atomic ratio of NiWO_4_ and Fe_0.5_Ni_0.5_WO_4_ in an alumina mortar with ethanol for wet mixing. The mixed powders underwent heat treatment in an electric box furnace at 900°C for 12 h. The crystal structure characterization of the synthesized powders was performed by X-ray diffraction (XRD, Smart Lab, Rigaku) with Cu Kα radiation and Raman spectroscopy (LabRAM ARAMIS, Horiba Jobin Yvon).

### 2.2 Theoretical calculation

The structural optimization and electronic structure calculations were performed using the density functional theory (DFT), as implemented in the QUANTUM-ESPRESSO (Sandro et al., 2005; Giannozzi et al., 2009; Giannozzi et al., 2017), using the Perdue–Burke–Ernzerhof (PBE) generalized-gradient approximation (GGA) functional (Perdew et al., 1996) and projector augmented wavefunction (PAW) pseudopotentials (Blöchl, 1994). Convergence tests on kinetic-energy cutoff and k-point density showed that the 120 Ry and 4 × 4 × 4 Monkhorst–Pack (Monkhorst and Pack, 1976) k-point grid are efficient and accurate enough. The crystal structure (materials project ID: mp-21179) was double along the a-axis to reflect that NiWO_4_ has an antiferromagnetic order. The structure was then relaxed to the remaining force, and stress can be less than 10–3 Ry/Bohr and 0.1 kbar. These calculations were performed under the collinear spin-polarized scheme where the spin quantization axis was fixed to the Cartesian *z*-axis, which is only ∼0.0131° off from the crystal c-axis. To consider the correlated nature of NiWO_4_ appropriately, the Hubbard U repulsion parameters on Ni-3d, Fe-3d, and W-5d were added according to the scheme of Dudarev et al. (1998). Further relaxation of forces and stresses due to the inclusion of Hubbard U parameters were not performed. The Hubbard parameters were calculated self-consistently using the density functional perturbation theory (DFPT), as implemented in the HP code (Timrov et al., 2018; Timrov et al., 2021; Timrov et al., 2022).

### 2.3 Evaluation of the gas-sensing performance

A two-probe electrode configuration was employed for the gas-sensing analysis. NiWO_4_ and Fe_0.5_Ni_0.5_WO_4_ powders were put on the gold electrodes positioned on an alumina substrate with 0.2 mL of ethanol on synthesized powders, and the powder was physically pressed to fix the powder on the substrate. The gas-sensing performance of the fabricated sensors was evaluated within a custom-built chamber equipped with mass flow controllers, maintaining a fixed flow rate of 500 standard cubic centimeters per minute using air as the carrier gas. The sensors were exposed to the target gas concentrations increasing up to 20 ppm for 1,000 s, followed by a recovery period in the air for 500 s at room temperature, 100, and 200°C. The resistance values in air (R_a_) and upon exposure to the target gases (R_g_) were recorded, and the sensor response (R_g_/R_a_) was determined by calculating the ratio of the resistance values in ambient air to under the target gas exposure conditions. Gas-sensing measurements were performed for various gases, including NO_2_, H_2_S, SO_2_, benzene, CO, ethanol, and acetone.

## 3 Results

### 3.1 Substitutionally located Fe in the Ni site for SCES NiWO_4_

**FIGURE 1:**
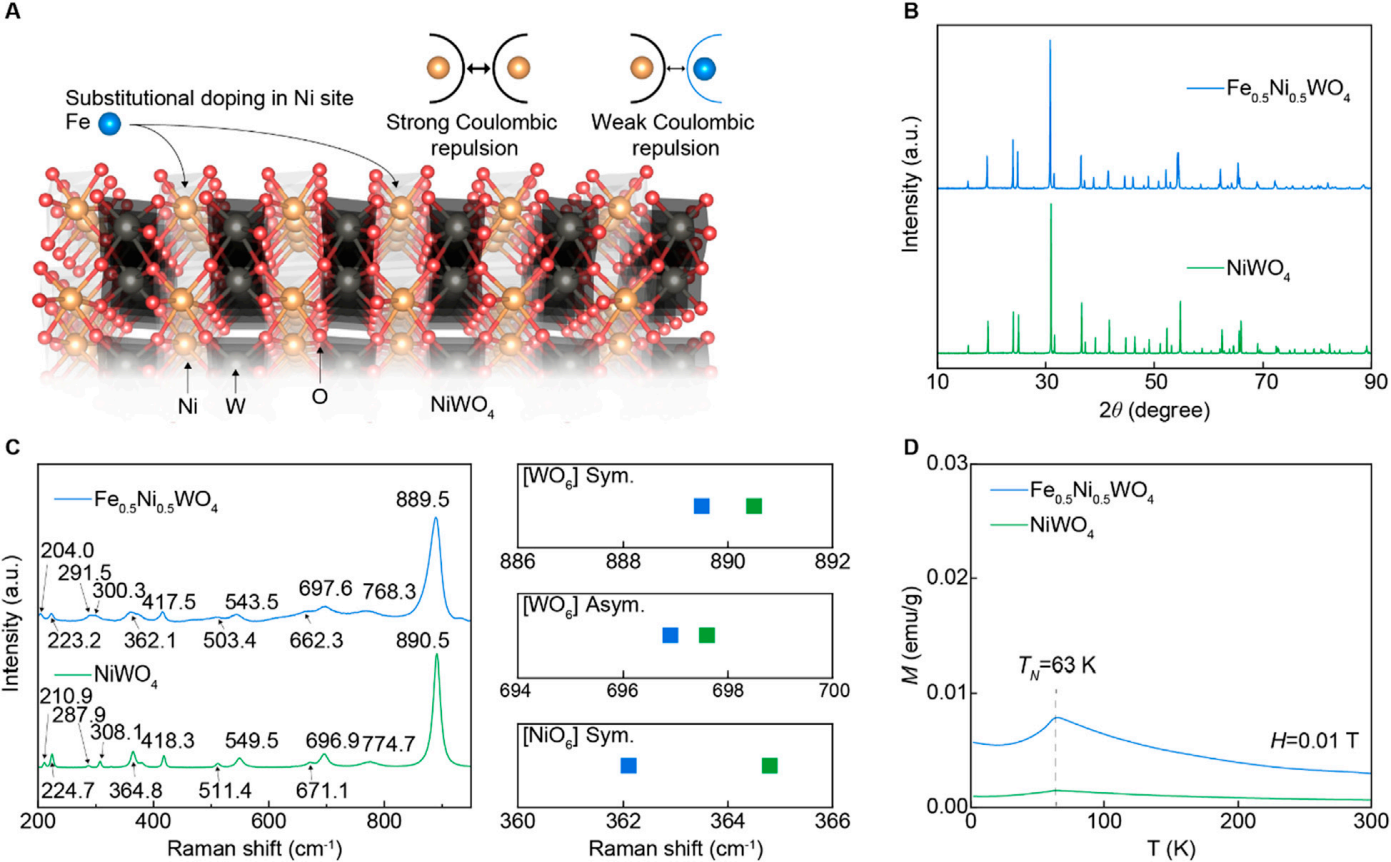
SCES insulator of Fe_0.5_Ni_0.5_WO_4_. **(A)** Schematic strategy of Fe cations in the NiWO_4_ crystal structure to modulate Coulombic repulsion. Difference in Coulombic repulsion between Ni–Ni and Ni–Fe. **(B)** XRD results of NiWO_4_ and Fe_0.5_Ni_0.5_WO_4_. **(C)** Raman spectroscopy results of NiWO_4_ and Fe_0.5_Ni_0.5_WO_4_. The plot on the right side is a description of the peak shifts for [WO_3_] symmetric, [WO_3_] asymmetric, and [NiO] symmetric vibration modes. **(D)** Temperature dependence magnetization curve under 0.01 T.

The NiWO_4_ strongly correlated electron system (SCES) exhibits a wolframite-type crystal structure (space group P2/c), composed of corner-shared [NiO_**6**_] and [WO_**6**_] octahedral sublattices, with significant Coulombic repulsion between Ni atoms (Prosnikov et al., 2017; Han et al., 2024; Suh et al., 2024). To modulate the Coulombic repulsion, we replaced Fe cations into the Ni sites of NiWO_4_ using a solid-state synthesis method, as depicted in Figure 1A. The Fe cations typically exhibit a +2 to +3 oxidation state and have an atomic radius of 1.26 Å, which is similar to that of Ni cations (1.26 Å; +2 to +3 oxidation state). In contrast, W cations have a larger atomic radius of 1.41 Å and an oxidation state of +5 to +6. The same wolframite-type structure of FeWO_4_, which also consists of corner-shared [FeO_6_] and [WO_6_] octahedral sublattices with analogous Wyckoff positions (Maignan et al., 2022), suggests that the Fe cations are substitutionally located in the Ni sites of NiWO_4_. Figure 1B shows that both the synthesized NiWO_4_ and Fe_0.5_Ni_0.5_WO_4_ powders exhibit a single phase. Raman spectroscopy results in Figure 1C reveal that these monoclinic structures display 36 distinct Raman vibrational modes, including 8 Ag and 10 Bg active modes. Notably, the Fe_0.5_Ni_0.5_WO_4_ sample shows a downward shift in peak positions compared to NiWO_4_, particularly in the Ni–O symmetric vibration mode within [NiO_6_], which shifts from 364.8 cm^−1^ in NiWO_4_ to 362.1 cm^−1^ in Fe_0.5_Ni_0.5_WO_4_, indicating weaker Ni–O bonding. This peak shift is more pronounced than those observed in the W–O asymmetric vibration mode near 698 cm^−1^ and the W–O symmetric vibration mode near 890 cm^−1^. The temperature-dependent magnetization (*M*) curve reveals a Néel temperature (*T*_N_) of 63 K for both NiWO_4_ and Fe_0.5_Ni_0.5_WO_4_ compositions, with Fe_0.5_Ni_0.5_WO_4_ exhibiting higher magnetization, as shown in Figure 1D, which suggests reduced Coulombic repulsion, which is common in SCES (Park et al., 2020). The XRD and Raman results confirm that Fe is located at the Ni site within the single phase of the NiWO_4_ SCES matrix.

**FIGURE 2:**
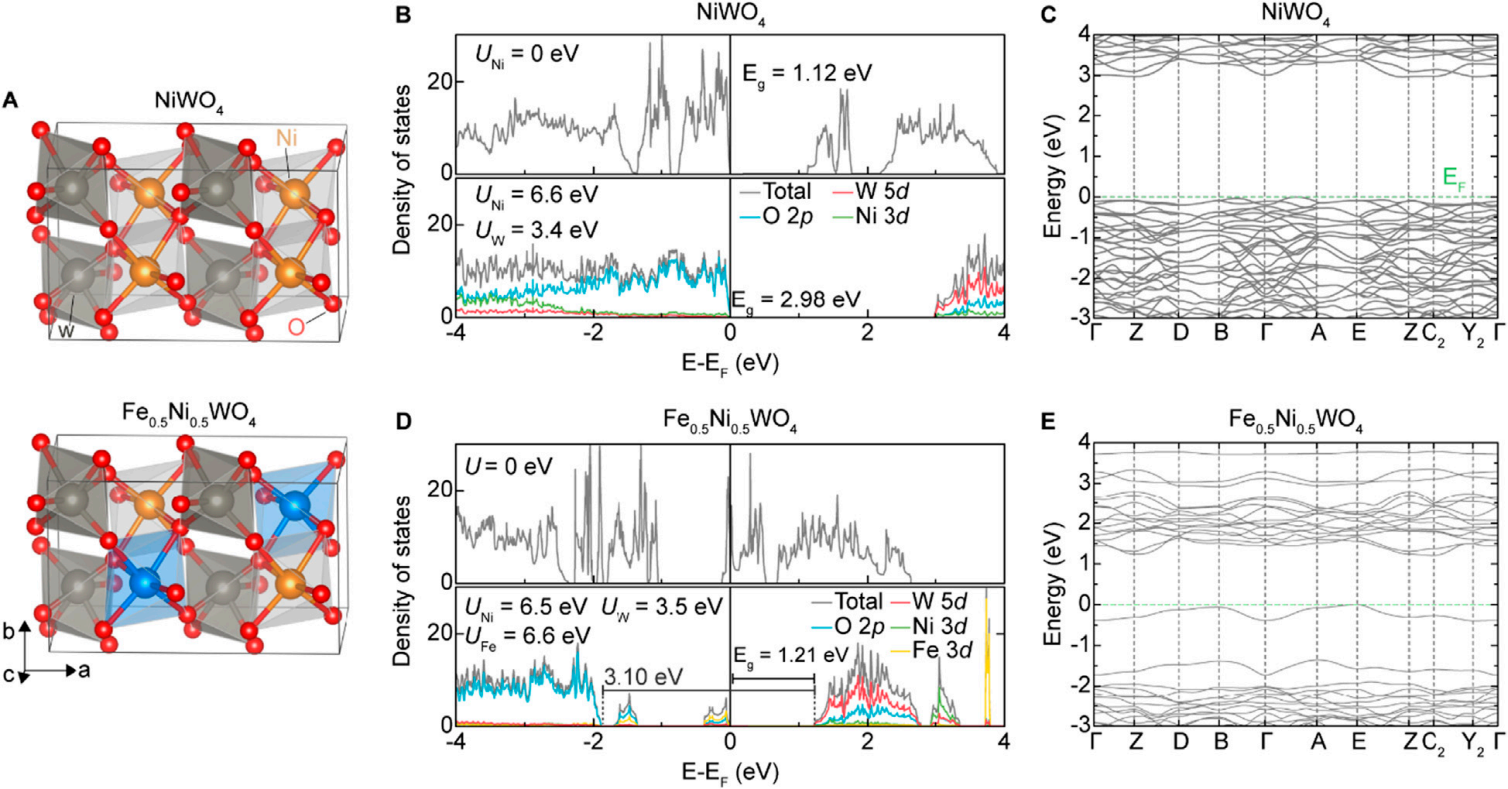
Theoretical results of NiWO_4_ and Fe_0.5_Ni_0.5_WO_4_ SCES. **(A)** Model structure of NiWO_4_ (top) and Fe_0.5_Ni_0.5_WO_4_ (bottom) from the DFT calculations. **(B, C)** Electronic structure of NiWO_4_ of DOS (*U* = 0) and PDOS plots (*U*_Ni_ = 6.6 eV; *U*_W_ = 3.4 eV) **(B)** and band structure with the considered *U* value **(C)**. **(D, E)** Electronic structure of Fe_0.5_Ni_0.5_WO_4_. The DOS and PDOS plot **(D)** and the band structure with the *U* value **(E)**.

To investigate the effect of Fe substitution theoretically, we calculated the electronic structure of NiWO_4_ and Fe_0.5_Ni_0.5_WO_4_ SCES, whose crystal structures are shown in Figure 2A. As reported in many earlier works, the Hubbard *U* scheme can enhance the accuracy of the calculation, especially the band gap. We first assumed 6.0 eV of on-site Coulomb repulsion energy for both Ni 3*d* and W 5*d*. The density functional perturbation theory (DFPT) calculation of Hubbard parameters was then performed on this ground state using a gamma-only ***q***-point mesh. This DFPT calculation yielded *U* = 6.5951 eV for Ni-3*d* and 3.4487 eV for W 5*d*. A new ground state with U = 6.6 eV for Ni-3*d* and *U* = 3.4 eV for W-5*d* was calculated. We again performed the DFPT calculation of Hubbard parameters, which resulted in quite converged values of *U* = 6.5727 eV for Ni-3*d* and *U* = 3.4561 eV for W-5*d*. Finally, we checked the dependency of the Hubbard parameters on the ***q-*** point mesh size. The DFPT calculations of *U* on the ground state with *U* = 6.6 eV for Ni-*3*d and *U* = 3.4 eV for W-5*d* were performed using ***q***-point mesh of sizes of 1 × 2 × 2 and 2 × 4 × 4. As the ***q***-point size increases, *U* parameters also increase, but it does not vary much, showing a difference of *U* less than ∼0.1 eV, as seen in Table 1, implying that the parameters are well-converged. The ground state with these self-consistently converged *U* parameters shows an indirect optical band gap of 2.98 eV, as shown in Figures 2B, C, which agrees well with some earlier results (Shepard and Smeu, 2018; Errandonea et al., 2023; Ye et al., 2023). We performed the same procedures to calculate the Hubbard parameters (Table 1), density of states (DOS), and the band structure of Fe_0.5_Ni_0.5_WO_4_ (Figures 2D, E). We supposed identical *U* values for two Ni atoms and two Fe atoms due to their structural symmetry. The converged values slightly differ from the value of NiWO_4_, but it rapidly converged, as iterated. The test for convergence on the ***q*** mesh revealed that the calculation with the gamma-point only has a small deviation. This deviation was reduced to less than 0.05 eV by an additional iteration. The correction with this converged *U* value modifies DOS remarkably, as shown in Figure 2D, showing that the system is strongly correlated. In addition, states mainly consisting of Fe-3*d* and O-2*p* appear near the Fermi energy. This contrasts that the states of NiWO_4_ near the Fermi energy mainly consist of Ni-3*d* and O-2*p* (Errandonea et al., 2023). These observations imply the remarkable alteration of the conduction mechanism and characteristic in Fe_0.5_Ni_0.5_WO_4_.

**TABLE 1:** Calculated Hubbard parameters of each atom in eV. The values on the right in each cell of *U* represent the calculated Hubbard parameter when DFPT is applied to the ground state of the value on the left.

Iteration	*U*_Ni_	*U*_Fe_	*U*_W_
*NiWO*_*4*_			
1	6.0 | 6.5951	—	3.4 | 3.4487
2	6.6 | 6.5727	—	3.4 | 3.4571
2, *q* = 1 × 2 × 2	6.6 | 6.6125	—	3.4 | 3.4668
2, *q =* 2 × 4 × 4	6.6 | 6.6143	—	3.4 | 3.4669
*Fe*_*0.5*_ *Ni*_*0.5*_ *WO*_*4*_			
1	6.9 | 6.3946	6.9 | 6.3772	3.5 | 3.4960
2	6.4 | 6.4320	6.4 | 6.4338	3.5 | 3.4718
2, *q* = 1 × 2 × 2	6.4 | 6.4731	6.4 | 6.5612	3.5 | 3.4865
2, *q* = 2 × 4 × 4	6.4 | 6.4740	6.4 | 6.5763	3.5 | 3.4875
3, *q* = 2 × 4 × 4	6.5 | 6.4651	6.6| 6.5522	3.5 | 3.4858

Summarizing the structure of Fe_0.5_Ni_0.5_WO_4_ from the experimental and theoretical results above, it maintains the wolframite-type crystal structure while modulating the Coulombic repulsion between Ni atoms, as evidenced by the crystal structure distortion and enhanced magnetic properties compared to NiWO_4_. Experimentally, the relatively higher Raman shift of the [NiO_6_] symmetric vibration mode, compared to the [WO_6_] vibration modes, indicates that Fe atoms preferentially occupy the Ni sites, leading to a distorted Ni–O bonding state. Additionally, the higher magnetization of Fe_0.5_Ni_0.5_WO_4_, in contrast to the antiferromagnetic phase of NiWO_4_, is attributed to the higher magnetic moment of Fe. Moreover, the presence of Fe states near the Fermi level (E_F_) and within a 3.1-eV large depletion region, as suggested by the DFT results, indicates improved carrier transfer compared to NiWO_4_. The following section demonstrates the emergence of gas-sensing performance in Fe_0.5_Ni_0.5_WO_4_, in contrast to the lack of gas-sensing functionality in NiWO_4_, as induced by the Fe dopant.

### 3.2 Emerging NO_2_ gas sensing in Fe_0.5_Ni_0.5_WO_4_

**FIGURE 3:**
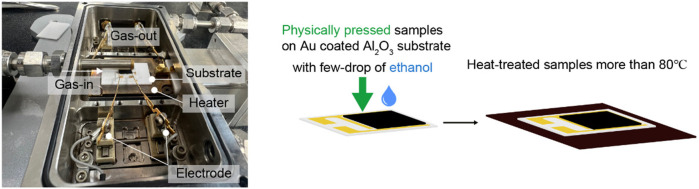
Photograph of the gas-sensing measurement system and the schematically described sampling methods.

**FIGURE 4:**
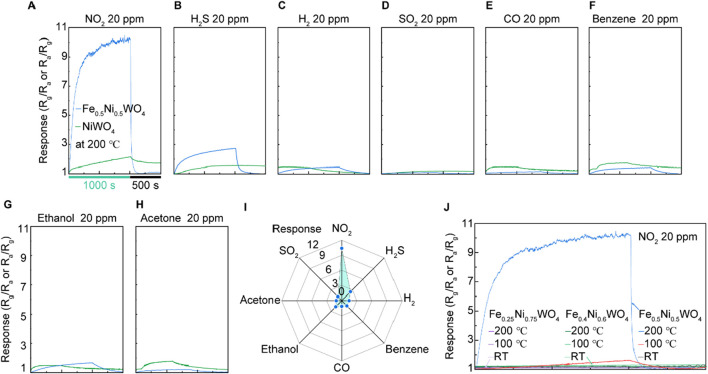
Gas-sensing results of NiWO_4_ and Fe_0.5_Ni_0.5_WO_4_ at 200°C under 20 ppm of different gases. **(A)** NO_2_ at RT, 100°C, and 200°C, respectively. **(B)** H_2_S at RT, 100°C, and 200°C. **(C)** H_2_. **(D)** SO_2_. **(E)** CO. **(F)** Benzene. **(G)** Ethanol. **(H)** Acetone. **(I)** Results of gas selectivity. **(J)** Comparison of the responses under an NO_2_ environment and different temperatures using Fe_0.25_Ni_0.75_WO_4_ and Fe_0.4_Ni_0.6_WO_4_.

The gas-sensing measurements were conducted using a two-electrode system, as depicted in Figure 3. The samples were prepared by pressing the synthesized powder onto an Au-coated Al_2_O_3_ substrate, followed by the addition of a few drops of ethanol. The samples were then heat-treated to vaporize the ethanol and secure the material onto the substrate. The gas sensing tests were performed under a 20-ppm concentration of various gases, including NO_2_, H_2_S, H_2_, SO_2_, CO, benzene, ethanol, and acetone, at room temperature, 100°C, or 200°C. The results for NO_2_ and H_2_S gas responses from Fe_0.5_Ni_0.5_WO_4_, shown in Figures 4A, B, indicate a significantly higher gas-sensing response at 200°C compared to that at RT and 100°C, highlighting this as the optimal operational temperature. A comparison of the gas-sensing responses between NiWO_4_ and Fe_0.5_Ni_0.5_WO_4_ reveals substantial differences in performance. For NO_2_ detection, Fe_0.5_Ni_0.5_WO_4_ exhibits a notable increase in resistance compared to the air environment, confirming that both Fe_0.5_Ni_0.5_WO_4_ and NiWO_4_ SCES function as p-type gas sensing materials. In contrast, NiWO_4_ samples consistently demonstrated a low and nearly uniform response, with an absolute value of gas sensing response (R_g_/R_a_ or R_a_/R_g_) close to 2 for all tested analyte gases, as shown in Figures 4A–H. However, Fe_0.5_Ni_0.5_WO_4_ displayed a significantly enhanced gas-sensing response, with an R_g_/R_a_ value of 10.4 under exposure to NO_2_ gas, marking it as the highest response among the tested gases. The second highest response was under H_2_S exposure, with a 2.8 response, which is still notably higher than the near 2 response observed for other gases such as H_2_, SO_2_, CO, benzene, ethanol, and acetone. The gas selectivity plot in Figure 4I further confirms the superior selectivity of Fe_0.5_Ni_0.5_WO_4_ for NO_2_. Figure 4J shows the comparison of NO_2_ responses at various Fe concentrations measured at different temperatures, including room temperature (RT), 100°C, and 200°C. Although Fe_0.5_Ni_0.5_WO_4_ exhibited a significant response of 11 at 200°C, Fe_0.25_Ni_0.75_WO_4_ and Fe_0.4_Ni_0.6_WO_4_ showed negligible gas-sensing responses across all measured temperatures. The higher gas-sensing performance of Fe_0.5_Ni_0.5_WO_4_ highlights the optimized Fe concentration and operating temperature as critical combined factors that enhance adsorption and facilitate carrier transfer between the gas molecules and the surface.

**FIGURE 5:**
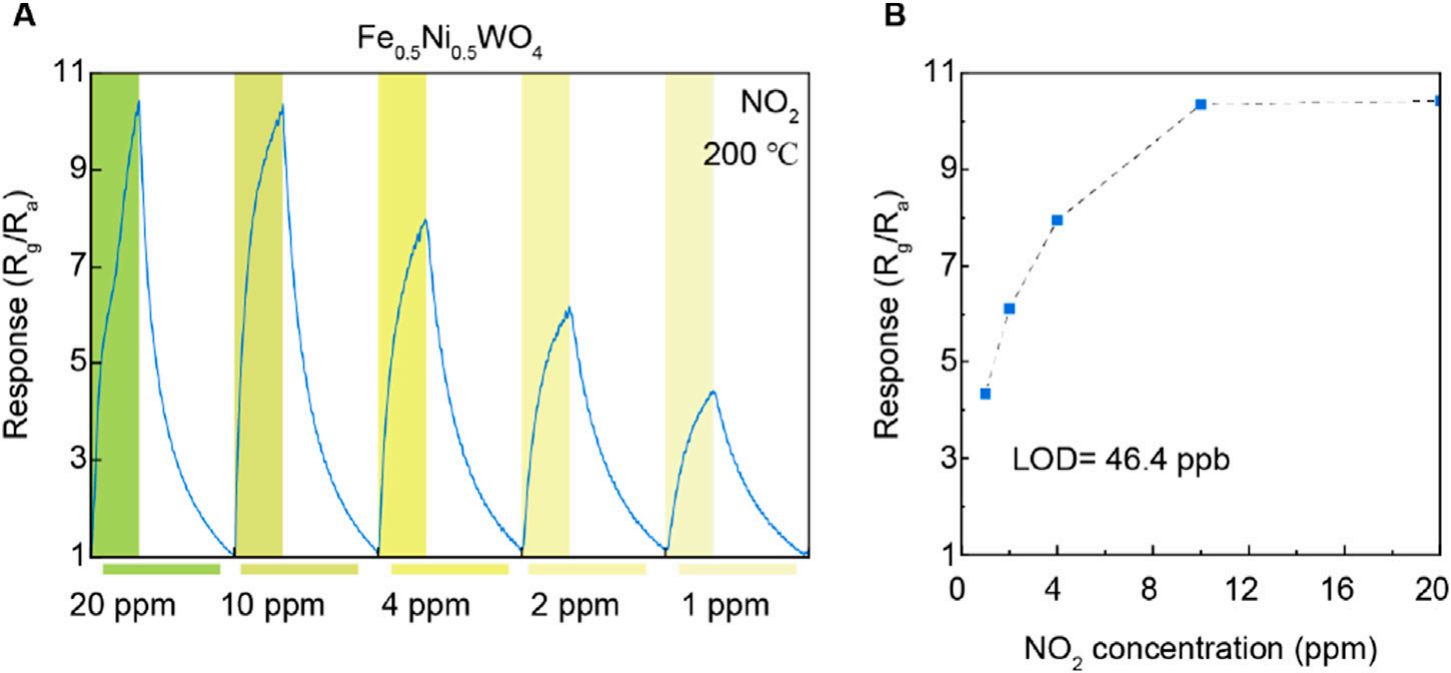
**(A)** Gas response under different NO_2_ concentrations. **(B)** Limit of detection for NO_2_ gases on Fe_0.5_Ni_0.5_WO_4_.

Furthermore, the gas-sensing response to varying NO_2_ concentrations is illustrated in Figures 5A, B. As the NO_2_ concentration decreases from 20 ppm to 1 ppm, a corresponding decline in gas response is observed, which is particularly noticeable between the concentrations of 10 ppm and 1 ppm. However, the response saturated at 10 ppm and 20 ppm of NO_2_, indicating a plateau in sensor performance at these levels. To further quantify the sensor’s sensitivity, we calculated the LOD based on the responses at NO_2_ concentrations of 10 ppm, 4 ppm, 2 ppm, and 1 ppm. From this analysis, an LOD of 46.4 ppb was determined. This value underscores the sensor’s capability to detect trace amounts of NO_2_ with significant precision. The ability to achieve such a low LOD highlights the effectiveness of Fe_0.5_Ni_0.5_WO_4_, which develops the material’s gas-sensing performance, particularly at lower concentrations. These results underscore the effectiveness of Fe doping in NiWO_4_, particularly in enhancing NO_2_ gas-sensing performance at 200°C. The distinct difference in response between NiWO_4_ and Fe_0.5_Ni_0.5_WO_4_ highlights the critical role of Fe cations in modulating the gas-sensing properties, suggesting that the Fe dopant level could serve as an activating agent for the gas adsorption site and improved the charge carrier transfer for selective gas-sensing materials and various chemistry applications.

## 4 Conclusion

The Fe dopant level located at band gap is used for enhancing the gas-sensing functionality on the NiWO_4_ SCES insulator, which shows negligible gas-sensitivity at 200°C for all measured gas molecules. The Coulombic repulsion between magnetic cations in NiWO_4_ open over 3.0 eV of the band gap, and dopant-level Fe is manipulated via being substitutionally located in the Ni site that developed 10.4 R_g_/R_a_ under 20 ppm of an NO_2_ environment. The Fe dopant implies playing an important role, such as activating the higher gas adsorption site or the higher carrier transferring from the charge carrier density changed between analyte gas molecules and materials' surface. These results encourage the use of further chemical sensing devices on SCES insulators by modulating the Coulombic repulsion between magnetic cations and dopant level-supporting at the Mott gap.

## Data Availability

The raw data in this manuscript will be available on request.
